# Identifying cow – level factors and farm characteristics associated with locomotion scores in dairy cows using cumulative link mixed models

**DOI:** 10.1371/journal.pone.0263294

**Published:** 2022-01-28

**Authors:** Andreas W. Oehm, Roswitha Merle, Annegret Tautenhahn, K. Charlotte Jensen, Kerstin-Elisabeth Mueller, Melanie Feist, Yury Zablotski

**Affiliations:** 1 Clinic for Ruminants with Ambulatory and Herd Health Services, Ludwig-Maximilians Universität Munich, Oberschleissheim, Germany; 2 Institute for Veterinary Epidemiology and Biostatistics, Freie Universitaet Berlin, Berlin, Germany; 3 Clinic for Ruminants and Swine, Faculty of Veterinary Medicine, Freie Universität Berlin, Berlin, Germany; 4 Clinic for Cattle, University of Veterinary Medicine, Foundation, Hannover, Germany; Michigan State University, UNITED STATES

## Abstract

Lameness is a tremendous problem in intensively managed dairy herds all over the world. It has been associated with considerable adverse effects on animal welfare and economic viability. The majority of studies have evaluated factors associated with gait disturbance by categorising cows into lame and non-lame. This procedure yet entails a loss of information and precision. In the present study, we extend the binomial response to five categories acknowledging the ordered categorical nature of locomotion assessments, which conserves a higher level of information. A cumulative link mixed modelling approach was used to identify factors associated with increasing locomotion scores. The analysis revealed that a low body condition, elevated somatic cell count, more severe hock lesions, increasing parity, absence of pasture access, and poor udder cleanliness were relevant variables associated with higher locomotion scores. Furthermore, distinct differences in the locomotion scores assigned were identified in regard to breed, observer, and season. Using locomotion scores rather than a dichotomised response variable uncovers more refined relationships between gait disturbances and associated factors. This will help to understand the intricate nature of gait disturbances in dairy cows more deeply.

## Introduction

Lameness in dairy cows continues to plague the global dairy production and generates a magnitude of challenges to professionals in the field. It often serves as an on-farm proxy for animal welfare [1, 2]. Lame animals experience severe, often chronic pain [2] which entails a profound incapacity to meet their potential performance level as well as the inability to express the broad range of their natural behavioural patterns. This includes considerable aberrations in feeding and lying behaviour which impedes cows from entirely satisfying their basic needs [3, 4]. Economic consequences of lameness are twofold and comprise expenditures from treatment costs and investments in control and preventive strategies as well as financial losses [5, 6]. The latter are indirect costs of lameness and include decreased milk yield [7, 8], impaired reproductive performance [9], and involuntary culling [10]. Lameness is characterised by aberrations in posture and gait that can be attributed to pain mostly due to claw pathologies [11]. In chronic cases, hyperalgesia is present irrespective of the severity of the underlying lesion. Even moderate changes in gait are associated with pain and hyperalgesia and lead to marked alterations in behavior, well-being and physiology [12–14]. An early identification and treatment of animals demonstrating impaired locomotion is hence indispensable in order to prevail serious infringements on animal health and welfare [15]. Lameness detection has commonly been based on the visual inspection of the animals under farm conditions [16]. An abundance of scoring approaches has been developed to describe the quality of dairy cow gait and posture. Sprecher et al. [17] have presented a frequently applied 5-point lameness scoring system that uses posture and gait to assess dairy cow locomotion. In biological sciences, observations are frequently recorded in an ordered manner with a finite set of categories [18]. This is especially the case for locomotion scores (LS) which represent a classic series of ordered categories representing ratings of gait disturbance [19]. Cows are usually regarded as lame when they obtain a certain score and the ordinal scale of the LS is hence dichotomised to create a binary outcome upon modelling [18]. This practice is yet affiliated with a considerable loss of information, precision and power [20–22]. Lameness is a classic example for a complex biological system of multifactorial aetiology, which covers a plethora of factors associated with housing conditions, management practises, and specific characteristics of the individual animal [23]. However, the number of studies on the associations of individual and environmental factors associated with different locomotion scores has been limited.

Cumulative link models (CLMs, syn. ordered logit models) provide a flexible and advanced regression approach to acknowledge the categorical, ordinal-scale nature of locomotion data [18, 24, 25]. To the best of our knowledge, reports on the implementation of ordered logit models in predictive modeling have been very limited [26]. The principal concept of ordered logit models is the proportional odds assumption, meaning that the relationship between any pair of the ordered, categorical target variable is the same [27–29]. Accordingly, the effects of the covariates are the same across all categories of the dependent variable. It has yet been discussed, that data frequently cannot satisfy the proportional odds assumption [27] and imposing proportionality may result in inconsistent estimation of outcomes [30]. In cases where discrete, categorical dependent variables not necessarily possess an ordered nature, multinomial models can be implemented for estimation of the outcomes [27, 31]. Hence, explanatory variables can be in distinct association with different levels of the response [27, 31, 32]. To our knowledge, O’Connor et al. [33] were among the first to characterise mobility quality by implementation of multinomial logistic regression. They evaluated associations between specific, categorical locomotion scores and cow-level factors. Randall et al. [34] used multinomial regression to analyse the association between BCS and lameness category where each cow was allowed to have repeated measures. It is yet important to understand that the ordered nature of data is not acknowledged in the multinomial approach. Moreira et al. [26] implemented an ordered logit model for locomotion scores upholding the proportional odds assumption for all factors in their study. In cases like the present study, partial proportional odds models represent a potent technique to address the problem that a subset of covariates may not exert the same effect on the different response levels while the ordered categorial scale of the response ought to be accounted for at the same time [27, 35, 36]. It is thus possible to relax the proportional odds assumption for certain variables which reject proportionality and allow them to differ by outcome or to be differently-ordinal. For instance, the difference of effects of a non-proportional covariate on the outcome may rather be a matter of degree or magnitude of the association [28]. This procedure ensures good model fit and efficient performance, flexibility as well as model parsimony [27, 36, 37]. Against this background, the aim of the present study was to build cumulative link mixed models on a large data set to identify and characterise animal- and husbandry-related factors associated with a higher locomotion score in dairy cows accounting for the ordered categorical nature of locomotion scores. As ordered logit model approaches and partial proportional odds models in particular have not been widely implemented, important insights can be gained into the complex setting of locomotion scores and potential risk factors for their increase.

## Material and methods

### Farm recruitment

Data were collected in the context of a large, cross-sectional study throughout Germany from December 2016 to August 2019. At the time this work was planned and conducted, prospective approval of this research by an animal research ethics committee was not necessary in Germany for this kind of study. Farms were selected via random sampling stratified by administrative district and farm size within the federal state using information from the national animal information data base (HIT) and from the Milchprüfring Bayern e.V. Within the study region, 1,250 farms thus were randomly selected in order to cover a response rate of at least 20%. Based on the farms registered in HIT, a region specific cut–off was determined so the range of different farm sizes within the study region could be represented (small: < 29 cows; medium: 30–52 cows; large: > 52 cows). Anonymity and data protection of contacted farms was ensured according to the German and European data protection legislation. The present study evaluated data from dairy farms throughout the south German federal state of Bavaria. Sampled farms received a letter containing information on the project. Enrolment was on a voluntary basis and interested farmers were to get in touch with the study team in order to arrange a date and time of the farm visit. Included farms were visited once between December 2016 and August 2019.

### On–farm data collection

Paper-based questionnaires and data entry forms were used to record data. These documents had been designed to cover a wide range of relevant fields including e.g. pasture management, flooring, free stall design and husbandry practises. Information on pasture access or/and an outdoor exercise area for cows and the operational type of the farm (organic vs. conventional dairy farming) were retrieved via personal interview with the farm manager. After each farm visit, collected data were manually transferred to a central SQL data base which allowed for plausibility checks and for the extraction of Microsoft Excel [38] data sheets. Type of flooring (solid, slatted), flooring surface (concrete, rubber) and free stall design were visually assessed. Floor slipperiness was assessed as follows [39, 40]: observers glided on the floor with their rubber boots to ascertain the extent of resistance: low slipperiness, moderate slipperiness, high slipperiness.

Data on breed, parity, milk yield, days in milk (DIM), milk constituents (milk fat, milk protein, and somatic cell count (SCC)) were retrieved from the national milk recording system (DHI) after written consent from the participating farms. Production data (milk yield, milk composition) are collected during a test day sampling once a month and were available up to 12 months prior to the farm visit.

Each cow was subjected to individual scoring. If more than 130 cows were present on a farm (n_farms_ = 3), a maximum of 130 cows were randomly scored. For example, if 260 cows were present on farm every second cow was to be scored. Consequently, out of ten cows, five cows were scored which resulted in the following scheme: one cow scored, one cow not scored, one cow scored, one cow not scored etc. Cows which escaped in the first place were scored later. Consistently, the most nearby cow was assessed. Attention was paid to maintain an even inclusion of feeding, resting, standing, and walking animals during the scoring procedure. Cows that were not scored were counted in order to deduce the correct number of cows present. All animals, both the scored as well as the ones not scored, were marked with a coloured spray specifically for this indication.

Body condition score (BCS) was assessed on the five point scale with 0.25 increment intervals established by Edmonson et al. [41]. Hock lesions were documented implementing a modified scoring method [42, 43]. Accordingly, hocks were assessed from a caudolateral view as follows: 1 = no skin change, 2 = hairless(visible loss of hair or hair breakage with skin shining through), 3 = swelling (presence of any sort of swelling without wound), 4 = wound (any form of disconnection of the skin, scab, no swelling), 5 = wound and swelling, 6 = no assessment possible due to solid plaque of manure on both hocks. Only the most severe of the present lesions was recorded. Leg and udder cleanliness were assessed according to Cook and Reinemann [44]: 1 = little or no manure, 2 = minor splashing, 3 = distinct plaques of manure, 4 = solid plaque of manure. Cows were assessed for locomotion using the scoring system by Sprecher et al. [17] that uses clear, objective descriptions of posture and gait characteristics to assign a LS to each cow between 1 (normal) and 5 (severely lame) with discrete 1.0 intervals: 1 = normal (level–back posture while standing and walking, normal gait pattern), 2 = mildly lame (level–back posture while standing, arched–back posture while walking, normal gait pattern), 3 = moderately lame (arched–back posture both while standing and walking, short–striding gait pattern with one or more limbs), 4 = lame (arched–back posture both while standing and walking, one deliberate step at a time during locomotion, favouring of one or more limbs), 5 = severely lame (additional display of extreme reluctance or inability to bear weight on one or more limbs). Cows were assessed from both a lateral and a caudal perspective when standing and walking at a normal pace in a straight path throughout the pen. Cows kept on pasture during the farm visit were not scored for locomotion.

### Data management

Microsoft Excel data sheets were exported from the central database. Plausibility of the data was checked automatically as well as by four co-authors assessing each variable’s distribution, also in regard to other variables. If implausible values were encountered, they were checked for both within the database (in order to detect potential irregularities during data extraction) as well as in the initial paper-based data entry forms and questionnaires (in order to detect potential incorrect introduction of recorded information in the database).

**Fig 1:**
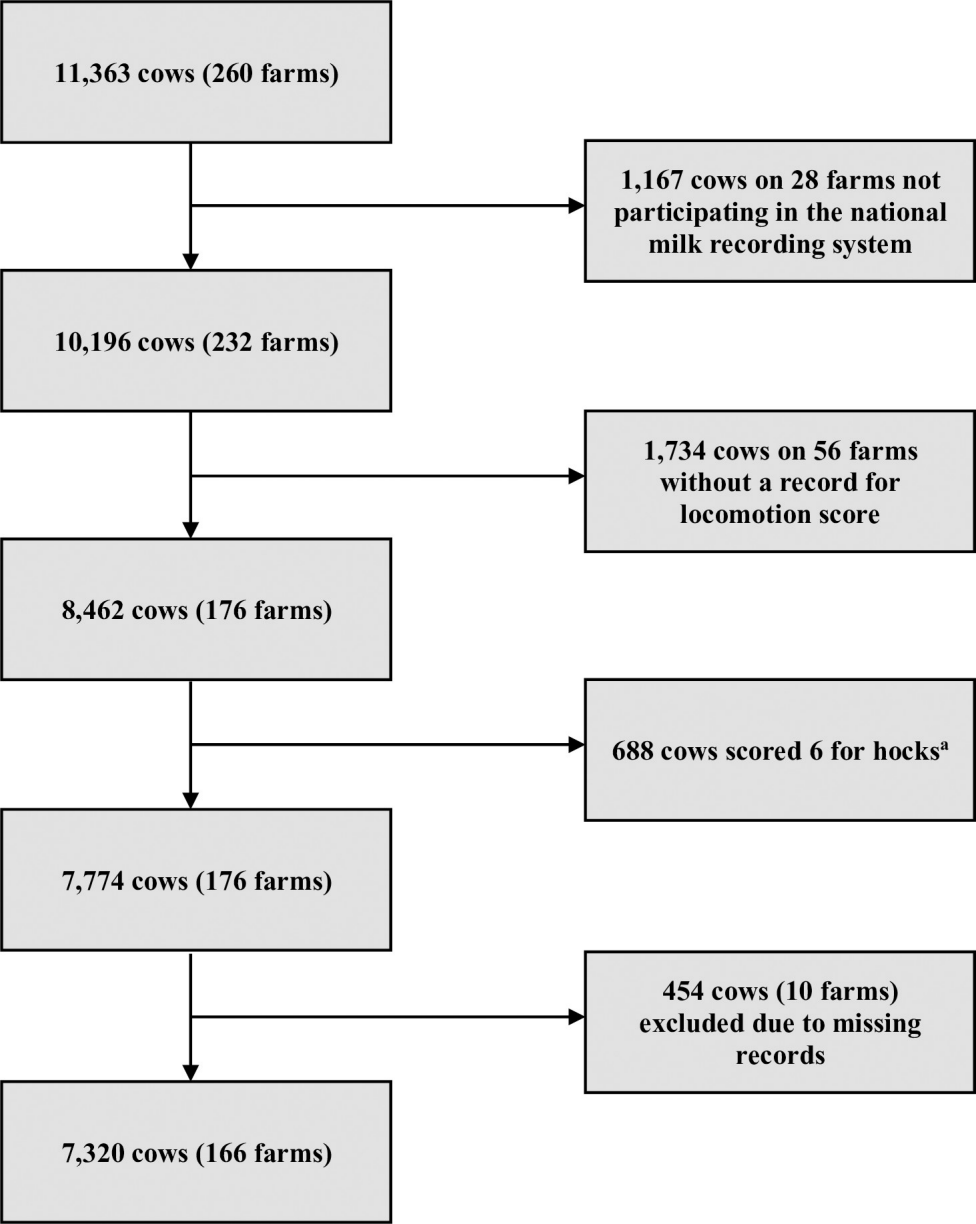
Flow chart displaying the workflow of creating the final data set for modelling from the original raw data. Out of 11,363 cows on 260 farms in the original data set, 28 farms (n_cows_ = 1,167) did not participate in the national milk recording system and were therefore excluded. A further 1,734 cows (n_farms_ = 56) were housed in tie stalls and in 688 cows no assessment of the hock region was possible due to solid plaques of manure on both hocks. Missing records led to the exclusion of 454 cows (n_farms_ = 10). Hence, a total amount of 7,320 cows on 166 farms were included in the current study. This final data set was used for all statistical analyses. n_cows_: absolute number of cows; n_farms_: absolute number of farms. ^a^ no assessment possible due to solid plaques of manure on both hocks.

The workflow of creating the final data set for the analysis is illustrated in a flowchart fashion (Fig 1).

The original data set contained data available for 11,363 cows on 260 farms. Cows housed on farms not enrolled in DHI (n_farms_ = 28) were removed from the data set (n_cows_ = 1,167) as well as cows without a record for LS (n_cows_ = 1,734). Cows which received score 6 for hocks (no assessment possible due to solid plaques of manure on both hocks) and observations with missing values (n_cows_ = 454) were excluded in order to create a complete cases data set for the analysis which consisted of a total amount of 7,320 observations on 166 farms.

As for BCS, cows were sorted into one of three BCS categories (underconditioned, normally/optimally conditioned, overconditioned) in alignment with their breed and stage of lactation [45–49]. An overview of the thresholds for the BCS categories is presented in S1 Table. *Parity* was further categorised into 1, 2, and ≥ 3. Since monthly information on milk yield, milk fat content, milk protein content, and SCC was available for each cow for a period of up to 12 months prior to the farm visit, individual animals may have had a number of one up to twelve different values for each of these three parameters. To obtain values that best and most plausibly reflect the individual animal, a Bayesian non-parametric bootstrap approach with 1,000 resamples with replacements was implemented to produce Bayesian medians for the variables milk yield, milk fat content, milk protein content, and SCC. This enabled us to create values that most plausibly reflected the overall production potential or level of the individual animal. SCC count was subsequently categorised based on the first and third quartile: < 50.35 (× 1,000) cells/ml, 50.35 (× 1,000) cells/ml to < 192.49 (× 1,000) cells/ml, ≥ 192.49 (× 1,000 cells/ml).

### Statistical analyses

All statistical analyses were performed in R software for statistical computing version 1.4.1103 [50, 51].

Moreover, we built univariable cumulative link models including a random effect for *farm* for each variable in regard to *LS* (S2 Table).

Cumulative link mixed models (ordered logit models) were fitted on cow-level with the clmm2() function from the ordinal package [52]. The outcome of interest was *LS* as an ordinal, categorical variable. One factor at a time was admitted to the model in a manual stepwise forward approach. To account for potential seasonality of lameness/locomotion events [53], *season* (spring: March, April, May; summer: June, July, August; autumn: September, October, November; winter: December, January, February) entered the model as a fixed effect. The importance of *farm size* (categorised according to distribution; small: < 44 cows; medium: 44–77 cows, large > 77 cows) and *observer* was acknowledged by adding both variables as fixed effects. A herd-specific random effect for *farm* was included in the model to correct for herd clustering. After each introduced variable, the Akaike’s Information Criterion was assessed. In order to comply with model parsimony [54], a manual stepwise backwards selection procedure was additionally carried out to address model complexity and to identify important factors [55, 56]. Each covariate (including the fixed effects *season*, *farm size*, and *observer*) in the final, forward selected model was due to a stepwise selection process and the model was assessed in the aforementioned manner.

The adaptive Gauss-Hermite quadrature method was implemented to compute the likelihood function. Hence, more accurate approximations of maximum likelihood estimates of the model parameters could be obtained compared with the Laplace approximation [57]. A number of 10 quadrature nodes was used. The identifiability of the model was assessed by checking the condition number of Hessian measures. In the present case the condition number of the Hessian measures did not suggest a problem with model identifiability. Large numbers (e.g. 10^5^ or higher) would imply ill definition as certain parameters remain unidentifiable. For all model parameters, profile likelihood confidence intervals (CI) set at 95% were calculated [58]. Odds ratios (OR) were produced to present the association between independent variables and outcome. In ordered logit model, odds ratios reflect the odds of locomotion score to increase by one point (e.g. from 1 to 2). CLMs conventionally assume proportional odds for model covariates which is why they are also referred to as *proportional odds models* [18, 27]. This assumption implies that for each explanatory variable within the model the estimates across the different levels of the response are the same between each pair of outcomes. The proportional odds assumption was checked in two consecutive steps to identify non-proportional odds structures among the variables: regarding each factor within the model, a separate cumulative link mixed model (CLMM) was built where the proportional odds assumption was relaxed (i.e. covariates were permitted to have non–proportional effects across the cumulative logits) for the respective variable. Subsequently, a likelihood ratio test of the proportional odds assumption was conducted to compare the model assuming proportional odds for all parameters with each model that relaxed the proportional odds assumption for one of the covariates [18, 27, 30]. If the proportional odds assumption is upheld, only a minor difference is detectable in the log-likelihood values of both models and the test is not statistically significant. The proportional odds assumption could not be adhered to by the covariates *breed*, *observer*, and *season*. To address this issue, the proportionality of odds was relaxed for these factors. A model relaxing the proportional odds assumption is referred to as a *partial proportional odds model* [27, 35]. Throughout the analysis, statistical significance was set at p ≤ 0.05.

(Multi-)collinearity was assessed creating variance inflation factors (VIFs). No evidence of (multi-)collinearity was confirmed as none of the VIFs exceeded a threshold value of 5 [59].

## Results

Descriptive statistics are presented in Tables 1 and 2.

**Table 1:** Animal-level descriptive statistics (number of cows per category and ratio) of categorical variables within the final, complete cases data set for analysis (7,320 cows on 166 farms).

Variable	Category	Counts (Number of cows)	%
Locomotion Score [17]	Normal	3012	41.15
	Mildly lame	2323	31.74
	Moderately lame	1244	16.99
	Lame	609	8.32
	Severely lame	132	1.80
Breed	Brown Swiss	711	9.71
	Holstein	580	7.92
	Simmental	5822	79.54
	other	207	2.83
Udder cleanliness [44]	Free of dirt	3127	42.72
	Slightly dirty	3032	41.42
	Moderately covered with dirt	911	12.45
	Covered with caked on dirt	250	3.41
Leg cleanliness [44]	Little/no manure	1251	17.09
	Minor splashing	3745	51.16
	Distinct plaques of manure	1877	25.64
	Solid/confluent plaques of manure	447	6.11
Observer	1	1340	18.31
	2	1351	18.46
	3	1815	24.80
	4	628	8.58
	5	1232	16.83
	6	568	7.76
	7	386	5.27
Season	Spring (March–May)	2286	31.23
	Summer (June–August)	2096	28.63
	Autumn (September–November)	1652	22.57
	Winter (December–February)	1286	17.57
Body condition score^a^ [45–49]	Underconditioned	832	11.37
	Optimally conditioned	4887	66.76
	Overconditioned	1601	21.87
Parity	1	2035	27.80
	2	1892	25.85
	≥ 3	3393	46.35
Type of flooring	Solid floor	2305	31.49
	Slatted floor	5015	68.51
Flooring surface	Concrete	6082	83.09
	Rubber	1238	16.91
Floor slipperiness [39, 40]	Low	3188	43.55
	Moderate	3251	44.41
	High	881	12.04
Free stall design	Mattress bedded free stall	2756	37.65
	Deep bedded free stall	4022	54.95
	Concrete free stall without bedding material	542	7.40
Hock lesions [42, 43]	No skin change	2616	35.74
	Hairless spot	3677	50.23
	Swelling (no wound)	339	4.63
	Wound (no swelling)	484	6.61
	Swelling and wound	204	2.79
Pasture access	Absent	5499	75.12
	Present	1821	24.88
Exercise access	Absent	5274	72.05
	present	2046	27.95
Farming type	Conventional	6522	89.10
	Organic	798	10.90
Farm size^b^	Small	1724	23.55
	Medium	3718	50.79
	Large	1878	25.66

^a^ categorised, cf. S1 Table.

^b^ categorised; small < 44 cows, medium 44–77 cows, large > 77 cows.

**Table 2:** Animal level descriptive statistics of continuous variables within the final, complete cases data set for analysis (7,320 cows on 166 farms).

Variable	Minimum	Mean	Standard deviation	Median	Interquartile range	Maximum
Days in Milk	0.00	196.80	119.70	189.00	184	835.00
Milk yield^a^	2.78	25.76	5.06	25.57	6.49	51.10
Milk fat^b^	2.40	4.21	0.46	4.18	0.58	7.70
Milk protein^b^	2.56	3.56	0.25	3.56	0.31	4.91
Somatic Cell Count^c^^,^ ^d^	10.00	167.30	231.84	95.55	142.14	6655.00

^a^ in kg.

^b^ in %.

^c^ × 1,000.

^d^ in number of cells / ml.

The mean farm size in the present study was 68 cows (lactating and dry animals) with a range from 11–231. Cows were housed on 65 small (< 44 cows), 77 medium (44–77 cows), and 24 large (> 77 cows) farms. German Simmental was the predominant cattle breed in the present study. As expected, gait disturbance was common both on animal as well as on farm level. The number and proportion of cows in the five different LS groups is presented in Table 1. Out of the 7,320 cows included in our study, 3,012 animals (41.15%) were assigned to LS 1 (normal gait and posture). In contrast, 4,308 cows (58.85%) of cows had signs of disturbed locomotion with 2,323 cows (31.75%) being scored 2 (mildly lame), 1,244 (16.99%) being scored 3 (moderately lame), 609 (8.32%) being scored 4 (lame), and 132 (1.80%) cows being assigned to score 5 (severely lame). Table 3 provides an overview of the prevalence of locomotion scores on farm level.

**Table 3:** Farm-level prevalence of locomotion scores across the 166 farms (7,320 cows) in the final, complete cases data set for analysis.

Locomotion Score [17]	Minimum	Mean	Standard deviation	Median	Interquartile range	Maximum
Normal	3.30	41.59	17.16	40.20	25.04	92.30
Mildly lame	8.80	32.05	9.93	31.20	14.45	66.70
Moderately lame	2.20	17.67	18.68	17.60	5.70	44.40
Lame	1.00	10.04	6.84	8.00	7.80	33.30
Severely lame	1.10	5.48	5.60	3.70	4.50	35.30

The results in Tables 4 and 5 stem from the final partial proportional odds model including 12 out of the 21 available variables.

**Table 4:** Final partial proportional odds model results for increasing locomotion score with covariates (cow level factors and farm characteristics) adhering to the proportional odds assumption.

Variable	Category	Estimate	Standard Error	Odds Ratio	95% Confidence Interval
Udder cleanliness [44]	Free of dirt	Reference	-	-	-
	Slightly dirty	0.17	0.05	1.18	1.07–1.31
	Moderately covered with dirt	0.40	0.08	1.48	1.28–1.72
	Covered with caked on dirt	0.33	0.13	1.40	1.09–1.79
Body condition score^a^ [45–49]	Optimal	Reference	-	-	-
	Overconditioned	-0.26	0.06	0.77	0.69–0.86
	Underconditioned	0.52	0.07	1.69	1.46–1.95
Hock lesions [42, 43]	No skin change	Reference	-	-	-
	Hairless Spot	0.35	0.06	1.42	1.27–1.59
	Swelling (no wound)	0.94	0.12	2.57	2.05–3.23
	Wound (no swelling)	0.90	0.10	2.47	2.02–3 02
	Swelling and wound	1.85	0.15	6.35	4.77–8.46
Parity	1	Reference	-	-	-
	2	0.47	0.06	1.61	1.42–1.83
	≥ 3	1.02	0.06	2.78	2.47–3.13
Somatic Cell Count^b^	1	Reference	-	-	-
	2	0.18	0.06	1.20	1.07–1.34
	3	0.25	0.07	1.29	1.12–1.48
Type of flooring	Solid	Reference	-	-	-
	Slatted	0.02	0.10	1.02	0.84–1.25
Flooring surface	Concrete	Reference	-	-	-
	Rubber	-0.17	0.12	0.84	0.66–1.07
Pasture access	Absent	Reference	-	-	-
	Present	-0 26	0.12	0.77	0.60–0.98
Farm size^c^	Small (< 44 cows)	Reference	-	-	-
	Medium (44–77 cows)	-0.08	0.12	0.94	0.75–1.18
	Large (> 77 cows)	-0.002	0.17	1.09	0.79–1.50

^a^ categorised; cf. S1 Table.

^b^ 1: < 50.35 × 1,000 cells/ml; 2: < 50.35 × 1,000 cells/ml to < 192.49 × 1,000 cells/ml, 3: ≥192.49 × 1,000 cells/ml.

^c^ categorised; small < 44 cows, medium 44–77 cows, large > 77 cows.

**Table 5:** Final partial proportional odds model results for increasing locomotion score and variables (breed, observer, season) with relaxed proportional odds.

Threshold	Variable (Category)	Estimate	Standard Error	Odds Ratio	95% Confidence Interval
-	Breed (Brown Swiss)	Reference	-	-	-
1 | 2	Breed (Holstein)	-0.46	0.17	0.63	0.45–0.89
2 | 3	Breed (Holstein)	-0.10	0.19	0.90	0.62–1.32
3 | 4	Breed (Holstein)	-0.08	0.26	0.93	0.56–1.54
4 | 5	Breed (Holstein)	-1.15	0.60	0.32	0.10–1.02
1 | 2	Breed (Simmental)	-0.42	0.14	0.65	0.50–0.86
2 | 3	Breed (Simmental)	-0.45	0.16	0.64	0.41–0.94
3 | 4	Breed (Simmental)	-0.47	0.21	0.62	0.41–0.94
4 | 5	Breed (Simmental)	-0.91	0.54	0.40	0.14–1.15
1 | 2	Breed (other)	0.27	0.20	1.31	0.87–1.94
2 | 3	Breed (other)	0.06	0.23	1.06	0.68–1.66
3 | 4	Breed (other)	0.22	0.34	1.24	0.64–2.40
4 | 5	Breed (other)	-0.19	0.80	0.83	0.17–3.97
	Observer (1)	Reference	-	-	-
1 | 2	Observer (2)	0.28	0.10	1.32	1.09–1.61
2 | 3	Observer (2)	0.03	0.11	1.04	0.84–1.28
3 | 4	Observer (2)	-0.15	0.15	0.86	0.64–1.17
4 | 5	Observer (2)	0.14	0.31	1.15	0.63–2.13
1 | 2	Observer (3)	0.59	0.10	1.81	1.49–2.19
2 | 3	Observer (3)	0.30	0.11	1.34	1.08–1.67
3 | 4	Observer (3)	0.13	0.16	1.14	0.84–1.54
4 | 5	Observer (3)	0.94	0.37	2.56	1.23–5.32
1 | 2	Observer (4)	-0.38	0.12	0.68	0.54–0.87
2 | 3	Observer (4)	-0.65	0.13	0.52	0.41–0.67
3 | 4	Observer (4)	-0.66	0.18	0.51	0.36–0.73
4 | 5	Observer (4)	0.05	0.41	1.05	0.47–2.36
1 | 2	Observer (5)	-0.35	0.11	0.70	0.57–0.87
2 | 3	Observer (5)	-0.39	0.11	0.67	0.55–0.83
3 | 4	Observer (5)	-0.64	0.15	0.53	0.39–0.70
4 | 5	Observer (5)	-0.45	0.28	0.64	0.37–1.10
1 | 2	Observer (6)	-0.20	0.14	0.82	0.62–1.07
2 | 3	Observer (6)	-0.46	0.14	0.63	0.48–0.83
3 | 4	Observer (6)	-0.47	0.19	0.62	0.43–0.90
4 | 5	Observer (6)	0.03	0.43	1.03	0.44–2.41
1 | 2	Observer (7)	0.12	0.18	1.13	0.80–1.61
2 | 3	Observer (7)	-0.30	0.19	0.74	0.51–1.08
3 | 4	Observer (7)	-0.65	0.25	0.52	0.32–0.84
4 | 5	Observer (7)	-1.23	0.39	0.29	0.14–0.62
	Season^a^ (autumn)	Reference	-	-	-
1 | 2	Season (spring)	0.14	0.15	1.15	0.85–1.54
2 | 3	Season (spring)	0.03	0.16	1.03	0.76–1.39
3 | 4	Season (spring)	0.13	0.18	1.14	0.81–1.61
4 | 5	Season (spring)	0.51	0.27	1.67	0.99–2.82
1 | 2	Season (summer)	-0.10	0.15	0.90	0.67–1.22
2 | 3	Season (summer)	-0.11	0.16	0.90	0.66–1.22
3 | 4	Season (summer)	-0.009	0.18	0.99	0.70–1.40
4 | 5	Season (summer)	0.52	0.28	1.68	0.97–2.92
1 | 2	Season (winter)	0.21	0.17	1.23	0.88–1.74
2 | 3	Season (winter)	0.10	0.18	1.10	0.77–1.57
3 | 4	Season (winter)	0.54	0.21	1.71	1.13–2.59
4 | 5	Season (winter)	1.04	0.34	2.82	1.45–5.48

^a^ spring = March–May; summer = June–August; autumn = September–November; winter = December–February.

In total, 9 out of the 12 variables entering the model adhered to the proportional odds assumption meaning that for each covariate the estimates across the different locomotion scores are the same between each pair of outcomes. Results cover 7,320 dairy cows on 166 farms. Low body condition, elevated somatic cell count, more severe hock lesions, increasing parity, absence of pasture access, and poor udder cleanliness were relevant variables associated with higher locomotion scores.

For the variables *breed*, *observer*, and *season* the proportional odds assumption was relaxed in the final model. Threshold estimates were generated for these covariates indicating that the outcome is different and not proportional across categories, i.e. the effect of the covariate depends on the threshold level of the response. These thresholds are interpreted separately for each level of locomotion score change. The model included 7,320 dairy cows on 166 farms.

The random effect, i.e. the variability between farms unexplained by the final CLMM was 33%. An increasing degree of dirt–staining of the udder was associated with higher locomotion scores: Compared with udders free of dirt, cows with slightly dirty udders (OR 1.18; CI 1.07–1.41), cows with udders moderately covered with dirt (OR 1.48; CI 1.28–1.72), and cows with udders covered with caked on dirt (OR 1.40; CI 1.09–1.79) had higher odds for a higher locomotion score. A low BCS, taking breed and DIM into account, entailed increased odds for a higher locomotion score compared with optimally conditioned cows (OR 1.69; CI 1.46–1.95). By contrast, a high BCS in regard to breed and DIM, i.e. overcondition, was associated with lower odds for a higher locomotion score (OR 0.77; CI 0.69–0.86). Hock lesions turned out to be a strong covariate associated with increased locomotion scores. Compared with cows without skin changes in the tarsal area, higher odds for increased LS were present in cows with a hairless spot (OR 1.42; CI 1.27–1.59). Both swelling without wound (OR 2.57; CI 2.05–3.23) and wound without swelling (OR 2.47; CI 2.02–3.02) quite similarly increased the odds for higher LS. More than six times the odds for a more severe LS were present in cows with swelling and wound at the hocks (OR 6.35; CI 4.77–8.46). Compared with first–lactation cows, increasing parity was associated with higher odds for a more severe LS both for parity 2 (OR 1.61; CI 1.42–1.83) and parity ≥ 3 (OR 2.78; CI 2.47–3.13). A higher SCC was associated with higher LS. Compared with animals within category 1 (< 50.35 (× 1,000) cells/ml), cows in categories 2 (50.35 (× 1,000) cells/ml to < 192.49 (× 1,000) cells/ml) and 3 (≥ 192.49 (× 1,000 cells/ml) experienced 1.20 times (CI 1.07–1.34) and 1.29 times (CI 1.12–1.48) the odds for a higher LS, respectively. Pasture access entailed lower odds for higher LS (OR 0.77; CI 0.60–0.98) compared with cows without access to pasture grounds.

Since the proportional odds assumption was relaxed for the three variables *breed*, *observer*, and *season* in the final model, threshold estimates were generated for these variables. This means that dependent of the form of the covariate, the outcome is different and not proportional across categories. These thresholds are interpreted separately for each level of change in LS, i.e. the effect of the covariate depends on the threshold level of LS. Table 5 provides a compilation of the final model results for all non-proportional variable categories in regard to the single target thresholds of LS (1|2, 2|3, 3|4, 4|5). Compared with Brown Swiss cows, Holstein cows had lower odds for a more extreme LS on both ends of the scale: 1|2 (OR 0.63; CI 0.45–0.89), 4|5 (OR 0.32; CI 0.10–1.02). The Simmental breed appeared to be of a more ordinal nature. Their effect varied in magnitude in regard to the LS thresholds indicating that a certain extent of ordinal nature can be contributed to Simmental although the odds are not proportional. Accordingly, Simmental cows experienced overall lower odds of having a lower LS compared with Brown Swiss: 1|2 (OR 0.65; CI 0.50–0.86), 2|3 (OR 0.64; CI 0.41–0.94, 3|4 (OR 0.62; CI 0.41–0.94), 4|5 (OR 0.40; CI 0.14–1.15). Expectedly, observer was associated with LS. A detailed compilation of the results for different observers is presented in Table 5. In general, some observers were associated with higher or lower odds for a lower LS, whereas others appeared to be associated with higher odds of assigning more extreme scores in both directions of the LS scale regarding severity. As for season, higher odds for a higher LS (4|5) were yielded for spring (OR 1.67, CI 0.99–2.82) compared with autumn. Similarly, higher odds for a higher LS (3|4, 4|5) were present for winter (OR 1.71, CI 1.13–2.59; OR 2.82, CI 1.45–2.82).

## Discussion

A dearth of evidence has been present on the association of animal–level and management–related factors with individual locomotion scores in dairy cows. To the best of our knowledge, this is the first time a complex partial proportional odds model was applied to evaluate factors related to housing, management, and the individual animal in regard to gait disturbance. This work is among the first to comprehensively collect data on dairy farms in Germany and in particular in the south German region of Bavaria as well as to evaluate them using inferential models.

When interpreting the results of our study, it is important to be aware of the cross-sectional nature of data collection which entails certain limitations specifically due to the study design [60]. Exposures as well as outcomes are assessed at the same time, which represents a potential source of bias in the way data are recorded. This is a shortcoming of the study design itself. Although we cannot rule out the introduction of bias at this stage, we are convinced to have minimised the risk by following clearly defined and comprehensive standard operating procedures that included repeated training of observers and internal discussions. Moreover, a large number of cows were included in the present analysis (n = 7,320), which enhances the chance for reliable inference. A second limitation of cross-sectional studies is that causal relationships cannot be deduced from the results and results require careful and appropriate interpretation. To infer causal patterns, future work ought to be specifically designed for this purpose. Another characteristic to be aware of is that participation in the present study was voluntary and interested farmers had to proactively get in touch with the study team. Farmer characteristics are pivotal in regard to the openness to external consultation and scientific knowledge [61, 62]. Therefore, proactive farmers who intrinsically are more inclined to implement innovative and evidence-based measures on their farms for the improvement of animal health and welfare may have been more likely to participate. Since these may represent farms with an overall improved health and welfare situation, the prevalence of more severe locomotion scores may be an underestimation and the true prevalences in the Bavarian dairy cow population may be higher. On the other hand, farmers with poor management, a higher prevalence of lameness and thus with cows with higher locomotion scores on their farms may have been more motivated to be enrolled as farms received a free consultation. In this case, the values reported in the current study may be an overestimation. We cannot entirely rule out a certain degree of selection bias in this context.

Bayesian medians for milk yield, milk protein content, milk fat content, and SCC were yielded by a Bayesian non-parametric bootstrap approach with 1,000 resamples with replacements. Non-parametric bootstraps allow for the estimation of parameters from a set of observations without necessitating assumptions of the distribution. For these four variables only, repeated measures were available for each animal within the data set up to 12 months prior to the LS assessment. Therefore, to render the data comparable to the remaining variables, the information needed to be conserved within a single value. Furthermore, the design of the data on milk yield, milk protein content, milk fat content and SCC was fairly unbalanced: For instance, some cows could have up to 12 values for each variable (given the absence of missing values) whereas other animals only had e.g. two, five or only one value. To conserve the highest level of information and to balance the data, the applied Bayesian bootstrap approach appeared to be an innovative, promising tool to yield the most realistic Bayesian median. The most prominent advantage of this tool is that the Bayesian bootstrap rather makes a likelihood statement about a parameter instead of a sole frequency statement [63]. Hence, the most plausible value could be deduced from the available data.

In our study, the proportionality of odds could not be met by the covariates *breed*, *observer*, and *season* in the final model, which appears logical as the distances between different severity levels of LS may not be consistently the same. As for observer, the proportional odds assumption would imply that different observers always assigned scores in an inherently identical and proportional manner in regard to LS severity. Yet, differences among observers as well as for the same have been well documented for locomotion scoring in dairy cattle. Therefore, the outcomes of this study for *observer* appear plausible as different observers tend to assign different levels of locomotion scores. Even with a small number of raters, consistent and reliable locomotion scoring remains a challenge [64]. This may lead to animals being assigned a score that does not reflect the reality. Hence, relevant associations may be mistaken during the modelling process and mistakable relationships may emerge. Calculation of inter-rater reliability for single locomotion scores was not possible in the present work as evaluation of observer agreement had been conducted for a dichotomised variable (‘lame/not lame’) throughout the cross-sectional study underlying this work and was not available for single locomotion scores. This might represent a limitation. To adjust for potential observer effects, *observer* was incorporated as a categorical covariate within the model. Interestingly, in the presence of *observer*, the other relevant factors remained significant, the model improved, and *observer* appeared as an additional important variable in regard to locomotion score. Following this procedure, we are convinced to have acknowledged the importance of *observer* in the context of locomotion scoring. Furthermore, based on these results, future studies evaluating single locomotion scores should consider the importance of *observer* by calculating measures of inter-observer reliability as well as by the inclusion of *observer* within the modelling procedure.

Dairy breeds share inherent differences, varying randomly in their manifestation, within as well as between breeds in regard to health [65]. Therefore, it appeared most plausible that the association between breed and LS was not of a proportional fashion. Compared with Brown Swiss cows, Holstein animals encountered lower odds for more extreme locomotion scores on both ends of the LS scale. This is an interesting finding, which yet lacks a simple explanation. Holstein cows are a potent dairy breed selected for maximum production and tend to partition more energy into milk rather than body reserves compared with other breeds [66, 67]. As a consequence, they may experience a more pronounced loss of body condition which has been associated with lameness [34]. Furthermore, dual–or multi–purpose breeds may have an improved stability of the locomotory apparatus due to their muscular build. Apart from that, Holstein cows have been described to be more susceptible to digital dermatitis (DD) compared with other breeds [68]. Higher locomotion scores, indicative of a greater severity of lameness, have been observed more frequently in cows with traumatic claw lesions than in cows with DD [69]. Furthermore, not all cows with DD have increased gait scores [70]. This background may lend support to the idea that Holstein animals in the present study were more frequently suffering from digital dermatitis which affected gait to the extent that neither very severe scores nor very mild scores were present but rather gait aberrations in the middle of the scale. As the underlying claw pathologies were not recorded in the present study, this requires further investigation.

The main dairy breed in the present study was German Simmental. This is a unique characteristic of our study as the body of evidence on Simmental cattle is limited [71]. Simmental cows are a dual purpose breed for dairy and beef production predominant in several regions [4, 72]. In our study, Simmental breed was associated with overall lower odds of a higher LS. We can only hypothesise about the true nature of this outcome as information is scant. As a dual purpose breed, Simmental cows may less efficiently partition energy and nutrients to milk production compared with classic dairy breeds, which tend to ruthlessly partition energy into milk rather than maintenance [66, 67]. Hence, a lower level of metabolic stress, a lower amount of body reserves being mobilised for milk production, and a less pronounced release of fat from the digital cushion may be present in Simmental cows. Therefore, the digital cushion may be more capable of maintaining its force dissipation properties. On the other hand, changes in posture, i.e. an arched back, may potentially be less easily detectable in Simmental cattle as they commonly have a stronger fundament, are shorter and stockier compared with Holstein cows. Moreover, Baird et al. [65] suggested that breeds may inherently differ in the way they move. Simmental cows may hence walk differently compared with Holstein. This might be a reason, why they were associated with a lower LS in our model. Grimm et al. [4] also applied the Sprecher scoring system on a herd consisting mainly of Simmental cows. Even though they slightly modified the original score in their study, they did not report difficulties in distinguishing different scores specifically in Simmental cattle. The locomotion scoring system implemented in our study was originally developed for Holstein cows [17]. It might hence be reasonable to consider potential inherent locomotive differences among breeds when scoring cows of breeds other than Holstein. This may require modifications of extant locomotion scoring approaches. To unravel the underlying nature of the association between locomotion score and breed in the present study, further research is necessary specifically in Simmental cows to gain insights to what extent results from studies in Holstein animals can be extrapolated to the Simmental breed.

The seasonality of lameness is a well-known aspect in epidemiology [53, 73]. Accordingly, it is plausible to imply an association with locomotion scores as well. Different seasons may not proportionally affect different locomotion scores but rather be associated with different levels of LS irrespective of potential proportionality. Season is a variable that does not represent a constant measure. It rather covers a variety of information including temperature, humidity, and other factors. Furthermore, information such as variations in feeding, pasturing, outdoor access or breeding management may share season specific characteristics and contribute to the information gathered within season. Moreover, season associated characteristics such as climate and weather conditions vary greatly in their intensity and duration not only between seasons but also within one season. Our results provide evidence for overall increased odds for a higher LS (thresholds 3|4 and 4|5) during winter compared with autumn. Furthermore, higher odds for a higher LS (threshold 4|5) were present in spring compared with autumn. No differences could be detected between summer and autumn with solely a tendency towards increased odds for a higher LS (threshold 4|5) in summer compared with autumn. These findings concur with those from other studies conducted in temperate regions. Lobeck et al. [74] reported a greater lameness prevalence both in winter and in spring compared with autumn, but no differences between summer and autumn. Hirst et al. [73] observed an increase of lameness prevalence during winter. Commonly, pasture access is limited or largely absent in winter. Furthermore, the use of footbaths is restricted due to freezing temperatures. This may give rise to infectious claw disorders such as digital dermatitis, which may hence result in higher locomotion scores. Moreover, potentially wetter conditions during the winter months may exert cumulative detrimental effects on overall claw soundness [53, 75, 76]. Additionally, cumulative effects during winter may increase locomotion scores in spring in a time delayed manner.

Globally, lameness represents a major challenge to the dairy industry as it impinges upon animal welfare and economic viability [77]. Lameness prevalence remains high even though great efforts have been undertaken to tackle the issue [78]. Gait disturbance was common among cows in the present data set which is in alignment with previous research [33, 79]. Only 41.15% of cows in our dataset had undisturbed posture and gait (LS 1). A further 31.74% of cows were mildly lame (LS 2, arched back during locomotion). Even though most studies applying the Sprecher scoring system tend to classify cows as lame with a LS ≥ 3 [71], we need to emphasise that a body of research has indicated that even a LS of 2 leads to considerable consequences for animal welfare and production [17, 80]. Score 2 is characterised by an arched back during locomotion. Yet not all cows arch their back due to lameness problems [81] and slight gait aberrations such as an arched back may as well be caused by claw trimming [82], milking frequency, gestational stage [83] or frictional properties of the floor [84]. Animals with slight aberrations in their gait pattern are difficult to detect, which is rendered even more challenging by the fact that cows tend to hide gait aberrations when observed. Since slight gait disturbances may lead to a prolonged period of pain and suffering, these cows ought to be identified and treated as early as possible [15].

Body condition has been demonstrated to be a potent factor associated with lameness related to claw horn lesions [34, 85]. From our results, we can infer that a low BCS is a strong factor associated with a more severe LS as well. Underconditioned cows experienced 1.69 times the odds for a higher LS compared with optimally conditioned animals. Body condition score has been positively associated with thickness of the digital cushion [86]. During periods of excessive weight loss, fat is mobilised from the digital cushion that hence loses much of its force dissipating capacities. Cows may develop impaired mobility as the decreasing dimensions of the digital cushion lead to increased pressures exerted on the corium, the germinative epithelium and the distal phalanx which promotes the development of traumatic claw lesions [87, 88]. This reasoning is further underscored by our result that overconditioned cows experienced 0.77 times lower odds for a higher LS compared with animals in optimal condition. Similar results were obtained by Moreira et al. [26] who reported that higher BCS was associated with better mobility. Overconditioning may ensure improved force dissipation within the digital cushion as enough fat is being disposed and more fat remains there when body reserves are mobilised. Associations between BCS and lameness in general have been outlined to be fairly intricate, as lameness itself enhances loss of body condition due to aberration in feeding behavior [4, 89]. There might be a perspective for future research particularly in regard to BCS and LS, as respective information has been scant.

Increasing parity was associated with higher odds of a more severe LS both for parity 2 and parities ≥ 3 compared with animals in their first lactation. This finding appears intuitive since older animals have been exposed to potential risk factors and previous lameness events for a longer time compared with younger herd mates [90]. Newsome et al. [91] have documented periostal exostoses in the plantar, axial, and abaxial aspects of the Tuberculum flexorium of cows with a history of claw horn disruption lesions. Furthermore, it has been illustrated that the composition and rigidity of the digital fat pads and the suspensory structures of the distal phalanx change with progressing age and around calving. The tensile strength and the supporting capacities of suspensory apparatus decrease and allow for an increased mobility of the distal phalanx [92–95]. This could be a potential explanation why dairy cows become more susceptible to claw horn disruption lesions with progressing age and why increasing parity was associated with higher LS.

More severe hock lesions were associated with higher LS. This is in alignment with previous research on the association of lameness with changes on the hock [72, 96]. The association between lameness and hock lesions is threefold: first, hock lesions, especially severe ones such as wounds, ulcerations or pronounced pressure sores cause pain and therefore may result in lameness [97]. As the majority of lameness cases are attributable to pathologies of the claws [11], the percentage of impaired mobility due to hock lesions may be major in some herds, yet not overall predominant. Second, lame cows often have difficulties rising and lying down. They alter their behaviour in order to mitigate pain from lameness causing conditions which may further impinge upon the integrity of their skin in the tarsal area as they are not capable of confidently lying down and rising and hence collide with elements of stall design [98, 99]. This is aggravated in an environment where bedding comfort is compromised as a result of unsatisfactory stall design, uncomfortable lying surface or wet or contaminated bedding material. Moreover, lame cows lie for a longer time [3] and therefore have a greater risk of developing hock lesions due to abrasion on rough stall surfaces, impeded blood flow as hocks may be exposed to prolonged levels of increased pressure, and irritation of the skin due to increased contact with soiled bedding [98, 99]. Third, lameness and hock lesions share a multitude of risk factors that facilitate their occurrence [99]. Hence, settings that increase the odds for lameness likewise promote the presence of hock lesions.

Leg dirtiness has been determined to be associated with infectious claw lesions [26]. Interestingly, the variable did not enter the final model. This might be a consequence of the exclusion of 688 animals where assessment of the hock was not possible due to the presence of solid plaques of manure on both hocks. The lower legs in these animals may have been severely covered with manure as well and the exclusion of these animals may have introduced a certain amount of bias which we cannot rule out. Yet udder cleanliness was associated with increasing LS. This may be due to the fact that udder dirtiness may reflect a more severe contamination of the animals. Dirt-covered udders may be indicative for faecal contamination of lying stalls. In a study in the northeastern United States and California, Chapinal et al. [100] demonstrated that an increase in the percentage of stalls with faecal contamination by 10% entailed higher odds for severe lameness. Dirt–stained udders may hence reflect a situation where clean lying areas are not available. They may also be indicative of overall poor barn hygiene. As lameness has been associated with prolonged lying periods [101], lame animals may prefer to defecate without rising which compromises udder cleanliness when changing lying position or rising eventually. Faecal contamination of stalls likely increases the bacterial load, which may promote infectious claw lesions and subsequently increase the odds of higher locomotion scores as observed from our results.

Elevated SCC increased the odds for a higher LS. Compared with cows in category 1, cows in categories 2 and 3 experienced higher odds for a higher LS. This is supported by recent results by O’Connor et al. [102] who reported an association between elevated SCC and impaired mobility. Higher SCC and impaired mobility are common findings in dairy cows and the combined presence of both conditions is not unusual [103]. In order to alleviate pain, lame cows needed longer resting periods compared with sound herd mates [101]. This may lead to increased exposure to pathogens at the teat end particularly if lying surfaces are poorly maintained. Contaminated udders which have appeared to augment the odds for a higher LS in the present study may be the link between an elevated SCC and a higher LS. Another aspect to be considered is an increased neutrophilic immune response in gait impaired dairy cows which may likewise result in higher SCC in those animals [104].

The presence of pasture access entailed lower odds for a higher locomotion score compared with the absence of pasture access for cows. This is underscored by the fact that cows were not assessed on pasture which could have falsified the locomotion scoring results and the results can be reliably attributed to the effect of general on-farm availability of pasture access itself. Grass provides optimal locomotory comfort to cattle. The soft, frictional properties of grass sod help cows increase their stride length and to walk confidently [105]. Weight is distributed more equally between both claws when walking on soft grounds as they can sink into the ground during locomotion rather than coming to a sudden halt as on inelastic, hard surfaces [106, 107]. Previous research has supported the hypothesis that pasture access is beneficial to locomotion and lameness dynamics [108]. In the light of the aforementioned, our results are in alignment with the existing literature.

Within Germany, large structural differences exist within dairy production which are characterised by different farm sizes, housing conditions, and management practices [109]. In Southern Germany, dairy farms are relatively smaller compared with other regions, e.g. East Germany, and mainly family–run businesses. Based on this information, the results of the present study can well be extrapolated to settings, where dairy farming is similar to the situation described in this work. Yet, conclusions can also be drawn for other backgrounds as outcomes are in alignment with previous work.

## Conclusions

The results from the present study incorporate the striking advantage that they come from a partial proportional odds model that acknowledged the ordered, categorical nature of locomotion scores. Hence, the association of the explanatory variables in the present model reflects the relation to increasing locomotion scores rather than classifications such as lame or not lame. This reduces the risk of misinterpretation and most importantly of loss of information and enables us to make inference about subtle changes in locomotion. The gain of information from including all levels of locomotion scores during modeling may well be a promising perspective to develop improved and concise preventive as well as therapeutic strategies. In a first step, this study evaluated factors associated with increasing LS. A future perspective should aim at identifying factors associated with single locomotion scores. This may well help detecting relevant differences in potential risk factors between locomotion scores and between the different degrees of severity regarding locomotion scores, respectively.

## Supporting information

S1 Table Categories of body condition score in regard to days in milk and breed used in the analysis. Under: underconditioned, Optimal: optimal body condition, Over: overconditioned.
(DOCX)

S2 Table Univariable cumulative link mixed models for increasing locomotion scores with all variables within the final dataset. (DOCX)

S1 File (PDF)

S2 File (PDF)

S3 File (PDF)

S4 File (PDF)

S1 Data (CSV)
