# Supplementary material for: Identifying cow – level factors and farm characteristics associated with locomotion scores in dairy cows using cumulative link mixed models
Source: PLoS One. 2022 Jan 28;17(1):e0263294. doi: 10.1371/journal.pone.0263294 (PMC8797239; doi:10.1371/journal.pone.0263294)
Supplement: S1 Table — Under: underconditioned, Optimal: optimal body condition, Over: overconditioned. (DOCX) [file pone.0263294.s001.docx]

S1 Table. Categories of body condition score in regard to days in milk (DIM) and breed used in the analysis [1-5].

| DIM | Breed | | | | | | | | |
| --- | --- | --- | --- | --- | --- | --- | --- | --- | --- |
|  | Holstein [2, 4, 5] | | | Brown Swiss [3-5] | | | Simmental/Other [1, 4, 5] | | |
|  | Under | Optimal | Over | Under | Optimal | Over | Under | Optimal | Over |
| 0 - 29 | ≤ 2.75 | 3.0 – 3.75 | > 3.75 | ≤ 2.75 | 3.0 – 3.75 | > 3.75 | ≤ 3.25 | 3.5 – 4.25 | > 4.25 |
| 30 – 99 | ≤ 2.5 | 2.75 – 3.25 | > 3.25 | ≤ 2.5 | 2.75 – 3.25 | > 3.25 | ≤ 3.0 | 3.25 – 4.0 | > 4.0 |
| 100 - 199 | ≤ 2.5 | 2.75 – 3.25 | > 3.25 | ≤ 2.5 | 2.75 – 3.25 | > 3.25 | ≤ 3.0 | 3.25 – 4.0 | > 4.0 |
| 200 – 299 | ≤ 2.75 | 3.0 – 3.75 | > 3.75 | ≤ 2.75 | 3.0 – 3.75 | > 3.75 | ≤ 3.25 | 3.5 – 4.25 | > 4.25 |
| > 300 | < 3.25 | 3.25 – 3.75 | > 3.75 | < 3.25 | 3.25 – 3.75 | > 3.75 | < 3.75 | 3.75 – 4.25 | > 4.25 |

Under: underconditioned, Optimal: optimal body condition, Over: overconditioned

References

1. Kritzinger F, Schoder G. Gesund und fit bringt optimale Leistung, BCS. Body Condition Scoring für Fleckvieh. Oberösterreichischer Tiergesundheitsdienst, Linz, Austria. 2009; pp. 1-2.

2. Kritzinger F, Schoder G. Gesund und fit bringt optimale Leistung, BCS. Body Condition Scoring für Holstein. Oberösterreichischer Tiergesundheitsdienst, Linz, Austria. 2009; pp. 1-2.

3. Kritzinger F, Schoder G, Mader C, Winckler R. Gesund und fit bringt optimale Leistung, BCS. Body Condition Scoring für Braunvieh. Tirol und Tiroler Tiergesundheitsdienst, Innsbruck, Austria; 2009.

4. Martin R, Mansfeld R, Hoedemaker M, de Kruif A. Milchleistung und Fütterung. In: de Kruif M, Hoedemaker M, Mansfeld R, editors. Tierärztliche Bestandsbetreuung beim Milchrind. 3rd ed. Stuttgart, Germany: Enke Verlag; 2014.

5. Heuwieser W, Mansfeld R. Beurteilung der Körperkondition bei Milchkühen, Teil 2. Milchpraxis. 1992; 30:10-4.
