# Supplementary material for: Identifying cow – level factors and farm characteristics associated with locomotion scores in dairy cows using cumulative link mixed models
Source: PLoS One. 2022 Jan 28;17(1):e0263294. doi: 10.1371/journal.pone.0263294 (PMC8797239; doi:10.1371/journal.pone.0263294)
Supplement: S2 Table — (DOCX) [file pone.0263294.s002.docx]

**S2 Table. Univariable cumulative link mixed models for increasing locomotion scores with all variables within the final dataset.**

| Predictor | Category | Estimate | Standard Error | Odds Ratio | 95 % Confidence Interval | P – value | Random Effect |
| --- | --- | --- | --- | --- | --- | --- | --- |
| Breed |  | | | | | | 0.47 |
|  | Brown Swiss | Reference | - | - | - | - |  |
|  | Holstein | 0.45 | 0.16 | 1.57 | 1.15 – 2.16 | 0.005 |  |
|  | Simmental | 0.55 | 0.14 | 1.74 | 1.33 – 2.27 | <0.001 |  |
|  | other | 0.10 | 0.18 | 1.10 | 0.77 – 1.57 | 0.60 |  |
| Days in milk | Continuous | -0.0004 | 0.0002 | 1.00 | 1.00 – 1.00 | 0.04 | 0.51 |
| Udder cleanliness [1] |  |  |  |  |  |  | 0.53 |
|  | Free of dirt | Reference | - | - | - | - |  |
|  | Slightly dirty | 0.35 | 0.05 | 1.42 | 1.29 – 1.57 | <0.001 |  |
|  | Moderately covered with dirt | 0.59 | 0.07 | 1.80 | 1.56 – 2.08 | <0.001 |  |
|  | Covered with caked on dirt | 0.54 | 0.13 | 1.71 | 1.34 – 2.18 | <0.001 |  |
| Leg cleanliness [1] |  | | | | | | 0.51 |
|  | Little/no manure | Reference | - | - | - | - |  |
|  | Minor splashing | 0.04 | 0.06 | 1.04 | 0.92 – 1.18 | 0.55 |  |
|  | Distinct plaques of manure | 0.11 | 0.08 | 1.12 | 0.96 – 1.29 | 0.14 |  |
|  | Solid/confluent plaques of manure | 0.31 | 0.11 | 1.36 | 1.09 – 1.69 | 0.006 |  |
| Observer |  | | | | | | 0.48 |
|  | 1 | Reference | - | - | - | - |  |
|  | 2 | -0.19 | 0.09 | 0.83 | 0.70 – 0.99 | 0.04 |  |
|  | 3 | -0.43 | 0.09 | 0.65 | 0.55 – 0.78 | <0.001 |  |
|  | 4 | 0.53 | 0.10 | 1.71 | 1.39 – 2.09 | <0.001 |  |
|  | 5 | 0.49 | 0.09 | 1.63 | 1.37 – 1.95 | <0.001 |  |
|  | 6 | 0.29 | 0.12 | 1.33 | 1.05 – 1.69 | 0.02 |  |
|  | 7 | 0.03 | 0.17 | 1.03 | 0.73 – 1.45 | 0.87 |  |
| BCS [2] |  | | | | | | 0.53 |
|  | Continuous | -0.45 | 0.05 | 0.64 | 0.58 – 0.70 | <0.001 |  |
| BCS^a^ (categorised) [3-7] |  | | | | | | 0.50 |
|  | Optimal | Reference | - | - | - | - |  |
|  | Underconditioned | 0.62 | 0.07 | 1.87 | 1.62 – 2.15 | <0.001 |  |
|  | Overconditioned | -0.20 | 0.06 | 0.82 | 0.74 – 0.92 | <0.001 |  |
| Hock lesions [8, 9] |  | | | | | | 0.33 |
|  | No skin change | Reference | - | - | - | - |  |
|  | Hairless Spot | 0.57 | 0.06 | 1.78 | 1.59 – 1.99 | <0.001 |  |
|  | Swelling (no wound) | 1.12 | 0.11 | 3.08 | 2.46 – 3.85 | <0.001 |  |
|  | Wound (no swelling) | 1.25 | 0.10 | 3.51 | 2.88 – 4.26 | <0.001 |  |
|  | Swelling and wound | 2.30 | 0.14 | 9.94 | 7.49 – 13.19 | <0.001 |  |
| Parity |  | | | | | | 0.59 |
|  | 1 | Reference | - | - | - | - |  |
|  | 2 | 0.49 | 0.06 | 1.64 | 1.45 – 1.85 | <0.001 |  |
|  | ≥ 3 | 1.14 | 0.06 | 3.11 | 2.78 – 3.48 | <0.001 |  |
| Season^b^ |  | | | | | | 0.49 |
|  | Winter | Reference | - | - | - | - |  |
|  | Spring | 0.04 | 0.18 | 1.04 | 0.73 – 1.48 | 0.88 |  |
|  | Summer | 0.34 | 0.18 | 1.41 | 0.99 – 2.01 | 0.06 |  |
|  | Fall | 0.23 | 0.20 | 1.26 | 0.86 – 1.85 | 0.23 |  |
| Milk yield |  | | | | | | 0.52 |
|  | Continuous | 0.01 | 0.005 | 1.01 | 1.00 – 1.02 | 0.06 |  |
| Milk fat |  | | | | | | 0.51 |
|  | Continuous | 0.05 | 0.05 | 1.05 | 0.95 – 1.17 | 0.30 |  |
| Milk protein |  | | | | | | 0.52 |
|  | Continuous | 0.35 | 0.09 | 1.43 | 1.19 – 1.71 | <0.001 |  |
| Somatic Cell Count |  | | | | | | 0.51 |
|  | Continuous | 0.0006 | 0.0001 | 1.00 | 1.00 – 1.00 | <0.001 |  |
| Somatic Cell Count (categorised) |  | | | | | | 0.52 |
|  | 50.35 ($\times$ 1,000) cells/ml | Reference | - | - | - | - |  |
|  | 50.35 ($\times$ 1,000) cells/ml to < 192.49 ($\times$ 1,000) cells/ml | 0.40 | 0.06 | 1.49 | 1.34 – 1.67 | <0.001 |  |
|  | ≥ 192.49 ($\times$ 1,000 cells/ml) | 0.62 | 0.07 | 1.86 | 1.64 – 2.11 | <0.001 |  |
| Type of flooring |  | | | | | | 0.46 |
|  | Solid | Reference | - | - | - | - |  |
|  | Slatted | 0.27 | 0.11 | 1.32 | 1.07 – 1.62 | 0.01 |  |
| Flooring surface |  | | | | | | 0.48 |
|  | Concrete | Reference | - | - | - | - |  |
|  | Rubber | -0.29 | 0.13 | 0.75 | 0.58 – 0.96 | 0.02 |  |
| Floor slipperiness [10, 11] |  | | | | | | 0.51 |
|  | High | Reference | - | - | - | - |  |
|  | Moderate | 0.04 | 0.15 | 1.04 | 0.78 – 1.38 | 0.81 |  |
|  | Low | -0.03 | 0.15 | 0.97 | 0.73 – 1.30 | 0.85 |  |
| Free stall design |  | | | | | | 0.44 |
|  | Mattress bedded free stall | Reference | - | - | - | - |  |
|  | Deep bedded free stall | -0.37 | 0.11 | 0.69 | 0.56 – 0.86 | <0.001 |  |
|  | Concrete free stall without bedding material | -0.11 | 0.19 | 0.90 | 0.62 – 1.31 | 0.57 |  |
| Pasture access |  | | | | | | 0.47 |
|  | Absent | Reference | - | - | - | - |  |
|  | Present | -0.45 | 0.13 | 0.63 | 0.49 – 0.82 | <0.001 |  |
| Exercise access |  | | | | | | 0.51 |
|  | Absent | Reference | - | - | - | - |  |
|  | Present | -0.14 | 0.14 | 0.87 | 0.66 – 1.14 | 0.31 |  |
| Farming type |  | | | | | | 0.48 |
|  | Conventional | Reference | - | - | - | - |  |
|  | Organic | -0.49 | 0.17 | 0.61 | 0.44 – 0.86 | 0.004 |  |
| Farm size^c^ |  | | | | | | 0.51 |
|  | Small | Reference | - | - | - | - |  |
|  | Medium | 0.004 | 0.13 | 1.00 | 0.77 – 1-31 | 0.98 |  |
|  | Large | 0.13 | 0.18 | 1.14 | 0.80 – 1.64 | 0.46 |  |

^a^ categorised; cf. Supplementary table S1

^b^ spring = March – May; summer = June – August; autumn = September – November; winter = December – February

^c^ small < 44 cows, medium 44 – 77 cows, large > 77 cows

References

1. Cook NB, Reinemann D. A tool box for assessing cow, udder and teat hygiene. 46th Annual Meeting of the National Mastitis Council; San Antonio, Texas, USA 2007; pp. 31 - 43.

2. Edmonson A, Lean I, Weaver L, Farver T, Webster G. A body condition scoring chart for Holstein dairy cows. J Dairy Sci. 1989; 72:68-78.

3. Kritzinger F, G S. Gesund und fit bringt optimale Leistung, BCS.Body Condition Scoring für Fleckvieh. Oberösterreichischer Tiergesundheitsdienst, Linz, Austria. 2009; pp 1-2.

4. Kritzinger F, Schoder G. Gesund und fit bringt optimale Leistung, BCS. Body Condition Scoring für Holstein. Oberösterreichischer Tiergesundheitsdienst, Linz, Austria. 2009; pp. 1-2.

3. Kritzinger F, Schoder G, Mader C, Winckler R. Gesund und fit bringt optimale Leistung, BCS. Body Condition Scoring für Braunvieh. Tirol und Tiroler Tiergesundheitsdienst, Innsbruck, Austria; 2009.

4. Martin R, Mansfeld R, Hoedemaker M, de Kruif A. Milchleistung und Fütterung. In: de Kruif A, Hoedemaker M, Mansfeld R, editors. Tierärztliche Bestandsbetreuung beim Milchrind. 3rd ed. Stuttgart, Germany: Enke Verlag; 2014.

5. Heuwieser W, Mansfeld R. Beurteilung der Körperkondition bei Milchkühen, Teil 2. Milchpraxis. 1992; 30:10-4.

8. Regula G, Danuser J, Spycher B, Wechsler B. Health and welfare of dairy cows in different husbandry systems in Switzerland. Prev Vet Med. 2004; 66:247-64.

9. Kielland C, Ruud LE, Zanella AJ, Osteras O. Prevalence and risk factors for skin lesions on legs of dairy cattle housed in freestalls in Norway. J Dairy Sci. 2009; 92:5487-96.

10. Bran JA, Costa JHC, von Keyserlingk MAG, Hotzel MJ. Factors associated with lameness prevalence in lactating cows housed in freestall and compost-bedded pack dairy farms in southern Brazil. Prev Vet Med. 2019; 172:104773.

11. Li KW, Chang W-R, Leamon TB, Chen CJ. Floor slipperiness measurement: friction coefficient, roughness of floors, and subjective perception under spillage conditions. Safety Sci. 2004; 42:547-65.
