# Supplementary material for: Identifying cow – level factors and farm characteristics associated with locomotion scores in dairy cows using cumulative link mixed models
Source: PLoS One. 2022 Jan 28;17(1):e0263294. doi: 10.1371/journal.pone.0263294 (PMC8797239; doi:10.1371/journal.pone.0263294)
Supplement: S1 File — (PDF) [file pone.0263294.s003.pdf]

Datum: ____________________ Betriebs ID: Lt. Datenbank

Studientierarzt: ____________________ Befragte Person: ____________________

Dieser Fragebogen beinhaltet Fragen zu allen möglichen Aspekten Ihres Betriebes.

Der Fragebogen ist standardisiert, damit man alle Betriebe vergleichen kann. Aus diesem Grund werde ich alle Fragen wortwörtlich vorlesen und möchte Sie bitten, diese kurz zu beantworten.
Wenn Sie eine Frage nicht verstehen, sagen Sie es mir bitte.

A. Betriebsstruktur

A1. Ist die Landwirtschaft Ihr Haupt- oder Nebenerwerb?

☐ **Haupterwerb** ☐ **Nebenerwerb** ☐ *weiß nicht* ☐ *k.A.*

A2. Wird dieser Betrieb konventionell oder ökologisch bewirtschaftet?

☐ **konventionell** ☐ **ökologisch** ☐ *weiß nicht* ☐ *k.A.*
