# Supplementary material for: Identifying cow – level factors and farm characteristics associated with locomotion scores in dairy cows using cumulative link mixed models
Source: PLoS One. 2022 Jan 28;17(1):e0263294. doi: 10.1371/journal.pone.0263294 (PMC8797239; doi:10.1371/journal.pone.0263294)
Supplement: S2 File — (PDF) [file pone.0263294.s004.pdf]

Date	Farm ID			
Interviewer	Interviewee			
This questionnaire contains questions about all potential aspects of this farm. The questionnaire is standardised in order to be able to compare farms. therefore, all questions will be read out loud exactly as they are written within the questionnaire. Please answer to them in a short manner. If a question remains unclear, feel free to tell.				
**A. Structure**				
A1. Is your farm the main source of income or a supplementary source of income				
main source	supplementary income	I do not know	not specified	
A2. Is this farm managed conventionally or according to organic farming principles?				
conventional	organic	I do not know	not specified	
