# Supplementary material for: Identifying cow – level factors and farm characteristics associated with locomotion scores in dairy cows using cumulative link mixed models
Source: PLoS One. 2022 Jan 28;17(1):e0263294. doi: 10.1371/journal.pone.0263294 (PMC8797239; doi:10.1371/journal.pone.0263294)
Supplement: S3 File — (PDF) [file pone.0263294.s005.pdf]

Date	Farm ID						
Interviewer							
Interviewee							
A. Pasture							
**A1. Is pasture access provided? If yes, which group of animals is pastured during which season?**							
	young stock	heifers	early lactating cows	cows in mid-lactation	cows in late lactation	early dry cows	cows in late dry period
no							
summer							
year round							
**A2. Is exercise area provided? If yes, which group of animals during which season?**							
	young stock	heifers	early lactating cows	cows in mid-lactation	cows in late lactation	early dry cows	cows in late dry period
no							
summer							
year round							
