# Supplementary material for: Identifying cow – level factors and farm characteristics associated with locomotion scores in dairy cows using cumulative link mixed models
Source: PLoS One. 2022 Jan 28;17(1):e0263294. doi: 10.1371/journal.pone.0263294 (PMC8797239; doi:10.1371/journal.pone.0263294)
Supplement: S4 File — (PDF) [file pone.0263294.s006.pdf]

Datum:

Betriebs ID: Lt. Datenbank

Prüftierarzt:

Befragte Person:

A. Weide-Haltung

A1. Kommen Ihre Tiere auf die Weide? Wenn ja, welche Gruppen zu welcher Jahreszeit?

	Jungvieh bis 1. Besamung	Tragende Rinder	LF	LM	LS	TSF	TSS
**Nein**	☐	☐	☐	☐	☐	☐	☐
**Sommer**	☐	☐	☐	☐	☐	☐	☐
**ganzjährig**	☐	☐	☐	☐	☐	☐	☐

A2. Haben Ihre Tiere einen Auslauf zur Verfügung? Wenn ja, welche Gruppen zu welcher Jahreszeit?

	Jungvieh bis 1. Besamung	Tragende Rinder	LF	LM	LS	TSF	TSS
**Nein**	☐	☐	☐	☐	☐	☐	☐
**Sommer**	☐	☐	☐	☐	☐	☐	☐
**ganzjährig**	☐	☐	☐	☐	☐	☐	☐
